# Women as Barbers

**Published:** 1875-06

**Authors:** 


### WOMEN AS BARBERS.

We shall never have clean barbers until the work which is expected of them is performed by women. It appears to be natural for most men to be unclean; offensive odors, and juices, and exhalations, seem to ooze from them naturally. Women appear to be cleaner, and sweeter, and purer, and the atmosphere about them is unquestionably more wholesome. This is easily accounted for. Men willingly defile themselves; women instinctively shrink from defilement. Men burn a horrible, poisonous plant in their mouths, or roll it under their tongues; women, worthy of the name, avoid the practice as they would any other unclean and offensive act. Men saturate themselves with dirty things, and many of them seem to delight in contact with them; women make a virtue of cleanliness, and endure foul contact unwillingly. These would appear to be good reasons why the trade of the barber should be given to women. Certainly there is nothing connected with it which is beyond their capacity; no labor as taxing and exhausting as thousands of working women and shopgirls now perform. Besides, it is work which pays; and there is no decent man in all the world who would not pay a trifle more for cleanly service from the deft and skillful hands of a fastidious woman than from an unwashed man, redolent of all foul smells. There is a fortune in store for a hundred good women in New York, who will open barber's shops and employ only women as helpers.

---

Out-door employment gives pleasure and health.

